# Small but mighty: cylindrical transparent cap-assisted cholangioscopic electrohydraulic lithotripsy and stone extraction for the treatment of difficult common bile duct stones

**DOI:** 10.1055/a-2729-2655

**Published:** 2025-11-14

**Authors:** Xin Li, Wei-Hui Liu

**Affiliations:** 1 89669 Department of Gastroenterology and Hepatology, Sichuan Provincial Peopleʼs Hospital, University of Electronic Science and Technology of China, Chengdu, China

Peroral cholangioscopy is gaining increasing recognition as a minimally invasive technique for managing common bile duct (CBD) stones, offering distinct advantages such as minimized trauma, fewer complications, and accelerated postoperative recovery 1 2 . In previous research, we introduced the application of a conical transparent cap to enhance selective biliary cannulation, termed as endoscopic retrograde cholangioscopy 3 4 . Building on this innovation, we fitted a cylindrical cap to the distal end of the cholangioscope, achieving enhanced intraoperative visualization, improved stone fragmentation positioning, and smoother stone extraction ( Video 1 ).

The cylindrical transparent cap facilitates cholangioscopic electrohydraulic lithotripsy (EHL) and subsequent extraction in treatment of impacted common bile duct stones.Video 1

A 58-year-old woman presented with challenging impacted CBD stones 1 month post-open choledocholithotomy. Endoscopic ultrasound revealed multiple stones, with the largest measuring 19.97 mm × 10.68 mm ( Fig. 1 ). Given the stone impaction, the presence of an indwelling T-tube, and biliary stenosis, we opted for a cholangioscopic approach as the safer alternative ( Fig. 2 **a** ). During the procedure, a cylindrical transparent cap was attached to the distal end of the cholangioscope ( Fig. 2 **b** ). This cap played a pivotal role: during electrohydraulic lithotripsy (EHL), it stabilized the target stone, preventing migration and potential ductal injury, while ensuring consistent probe-to-stone contact for efficient fragmentation ( Fig. 2 **c** ). Additionally, it protected the cholangioscope lens from bubbles and debris. For stone retrieval, the cap maintained a safe working distance between the retrieval basket and the lens, providing ample workspace and an unobstructed field of view ( Fig. 2 **d** ).

**Fig. 1:**
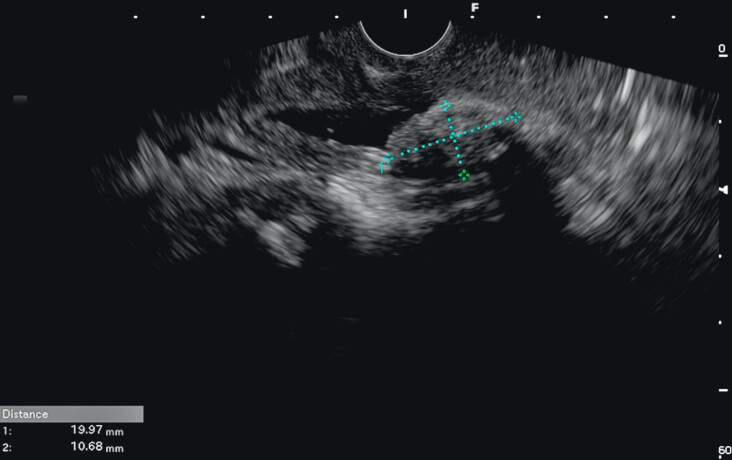
Endoscopic ultrasound showing dilation of the CBD with multiple stones, the largest measuring 19.97 mm × 10.68 mm. CBD, common bile duct.

**Fig. 2:**
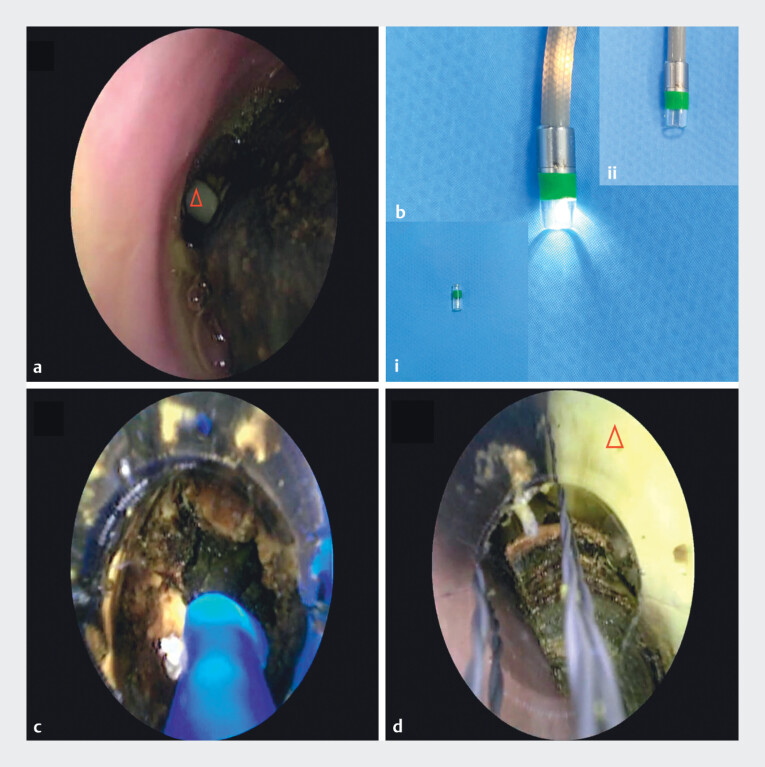
Cylindrical transparent cap-assisted cholangioscopic EHL and stone extraction for the treatment of difficult CBD stones. **a** Cholangioscopy reveals impacted stones in the CBD and an indwelling T-tube, resulting in a narrow operative space. **b** A cylindrical transparent cap is mounted onto the distal end of the cholangioscope. i. Transparent cap. ii. Transparent cap mounted onto the distal end of the cholangioscope. **c** During EHL, the cylindrical transparent cap stabilizes the stones, protects the field of view, and prevents damage to the endoscope and bile duct wall. **d** During stone retrieval, the cylindrical transparent cap maintains a safe working distance between the retrieval basket and the cholangioscope lens, ensuring ample workspace and a consistently clear field of view, while protecting the lens from bubbles, debris, and interference from the indwelling T-tube. Note: The red triangle marks the T-tube. The red triangle marks the T-tube. CBD, common bile duct; EHL, electrohydraulic lithotripsy.

In contrast to previous cholangioscopy-guided lithotripsy procedures performed without a transparent cap, the cylindrical transparent cap proved indispensable, serving multiple functions. On the one hand, during lithotripsy, it stabilized the stones, preserved the field of view, and prevented damage to the endoscope and bile duct wall; on the other hand, during stone extraction, it ensured an effective working field of view and expanded the outlet.

Endoscopy_UCTN_Code_TTT_1AR_2AH

## Notes

Endoscopy E-Videos https://eref.thieme.de/e-videos
*E-Videos* is an open access online section of the journal *Endoscopy* , reporting on interesting cases and new techniques in gastroenterological endoscopy. All papers include a high-quality video and are published with a Creative Commons CC-BY license. Endoscopy E-Videos qualify for HINARI discounts and waivers and eligibility is automatically checked during the submission process. We grant 100% waivers to articles whose corresponding authors are based in Group A countries and 50% waivers to those who are based in Group B countries as classified by Research4Life (see: https://www.research4life.org/access/eligibility/ ). This section has its own submission website at https://mc.manuscriptcentral.com/e-videos .
